# Supplementary material for: Ganglioside GD2 Enhances the Malignant Phenotypes of Melanoma Cells by Cooperating with Integrins
Source: Int J Mol Sci. 2021 Dec 31;23(1):423. doi: 10.3390/ijms23010423 (PMC8745508; doi:10.3390/ijms23010423)

Supplementary data:

Lists
1. Materials and Methods:
  EMARS/MS: Detailed procedures of MS analysis

2. Supplementary Table S1. Results of mass spectrometry of EMARS reaction

3. Figure legends for Supplementary figures:

(3-1). Supplementary Figure 1. Establishment of cell lines expressing ganglioside GD2.

(3-2). Supplementary Figure 2. Effects of anti-GD2 mAbs 3F8 on cell adhesion of GD2+ cells.

(3-3). Supplementary Figure 3. Results EMARS/MS analysis. Detection of integrin β1.

(3-4). Supplementary Figure 4. Results of MS analysis to identify tyrosine-phosphorylated bands, EGFR and FAK.

1. Marials and Methods

EMARS/MS: Detailed procedures of LC-MS/MS analysis

The trypsin-digested peptide samples were analyzed using mass spectrometer (LTQ Orbitrap velos, Thermo Scientific) coupled with nano-LC (EASY-nLC II, Thermo Scientific). The peptides were desalted and concentrated on the trap column (Capillary Ex-Nano Mono cap C18 Trap Column, 0.075 mm i.d. × 50 mm, GL sciences), and were then separated on an analytical column (Capillary Ex-Nano Mono cap C18 Nano-flow, 0.075 mm i.d. × 150 mm, GL sciences). For LC conditions, the following gradient was used (A, 0.1% formic acid/H2O; B, 70% MeCN, 0.1% formic acid in H_2O): 0 min 0% B; 0–2 min 0-29% B; 2–62 min 29-85% B; 62–67 min 85-100% B, 67–72 min 100% B, 300 nl/min. A top ten data-dependent acquisition MS method was used.

2. Supplementary Table S1. Results of mass spectrometry of EMARS reaction products

Accession	Molecules	S1 (mAb-treated)	S1 (mAb non-treated)
P08670	Vimentin	+	+
P35579	Myosin-9	+	+
P08238	Heat shock protein HSP 90-beta	+	+
P60709	Actin, cytoplasmic 1	+	+
P02768	Serum albumin	+	+
P68104	Elongation factor 1-alpha 1	+	+
P07900	Heat shock protein HSP 90-alpha	+	+
Q9BQE3	Tubulin alpha-1C chain	+	+
P15880	40S ribosomal protein S2	+	+
Q00610	Clathrin heavy chain 1	+	+
Q6IPR3	tRNA wybutosine-synthesizing protein 3 homolog	+	+
Q9Y490	Talin-1	+	+
Q5M775	Cytospin-B	+	+
P14618	Pyruvate kinase isozymes M1/M2	+	+
P13639	Elongation factor 2	+	+
Q6UWP8	Suprabasin	+	+
P60842	Eukaryotic initiation factor 4A-I	+	+
P05161	Ubiquitin-like protein ISG15	+	+
P05388	60S acidic ribosomal protein P0	+	+
P81605	Dermcidin OS=Homo sapiens	+	+
P62805	Histone H4 OS=Homo sapiens	+	+
O95433	Activator of 90 kDa heat shock protein ATPase homolog 1	+	-
P42224	Signal transducer and activator of transcription 1-alpha/beta	+	-
P05556	Integrin β1	+	-
P0DOX5	Immunoglobulin gamma-1 heavy chain	+	-
Q9BZF9	Uveal autoantigen with coiled-coil domains and ankyrin repeats	+	-
P26641	Elongation factor 1-gamma	+	-
Q09MP3	RAD51-associated protein 2	+	-
Q9NU22	Midasin	+	-
Q86YZ3	Hornerin	+	-
A6NIK2	Leucine-rich repeat-containing protein 10B	+	-
P60660	Myosin light polypeptide 6	+	-
P0CG48	Polyubiquitin-C	+	-
Q9BRP8	Partner of Y14 and mago	+	-

3, Legends for Supplementary Figures:

(3-1) Supplementary Figure 1. Establishment of cell lines expressing ganglioside GD2.

Expressions of gangliosides GD2 in GD2+ (S1 and S6) and GD2- (V4 and V9) cells were analyzed by flow cytometry using anti-GD2 mAb 220-51 and 3F8. Results by 3F8 were shown.

(3-2) Supplementary Figure 2. Effects of anti-GD2 mAbs 3F8 on cell adhesion of GD2+ cells. Effects of the treatment with anti-GD2 mAb 3F8 on cell adhesion were analyzed by the RT-CES system using GD2+ S1 and S6 cells and GD3- V4 and V9 cells in collagen I-precoated (5 μg/mL in PBS, 100 μL/well) 16-well microplates. Cells (1 x 10^4) were seeded in each well of the plates containing 200 μL culture medium. Purified anti-GD2 mAb 3F8 was added (10 μg/mL) to the wells at 3.0 h after starting cell adhesion measurement. Antibody-treated cells are indicated as `T`.

(3-3) Supplementary Figure 3.

Detection of integrin β1 in MS.

A peptide of integrin β1 RDNTNEIYSGK was found only in mAb+ sample (*right*), and results of MS/MS analysis of fragment ions (*left*). Enlarged images were separately shown for better resolution.

(3-4) Supplementary Figure 4.

Ten peptides of EGFR can be detected in the upper gel as listed (top).

Distribution of those peptides (cover map) in EGFR molecule (green) was shown (bottom).

Fragment ion patterns of the individual peptides of EGFR (EGFR1~10) are shown.

Six peptides of FAK can be detected in the upper gel as listed (top).

Distribution of those peptides (cover map) in FAK molecule (green) (bottom) was shown (bottom).

Fragment ion patterns of the individual peptides of FAK (FAK1~6) are shown.

Supplementary Figure S1.

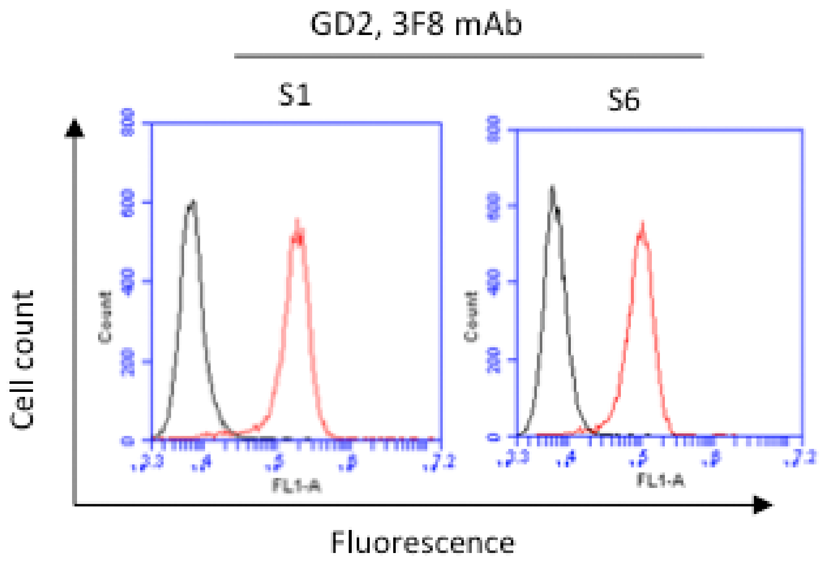

Supplementary Figure S2.

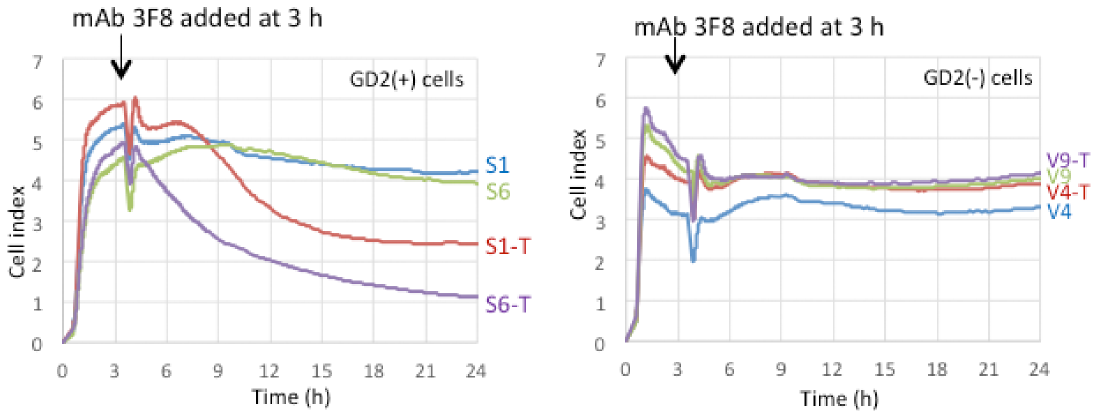

Supplementary Figure S3.

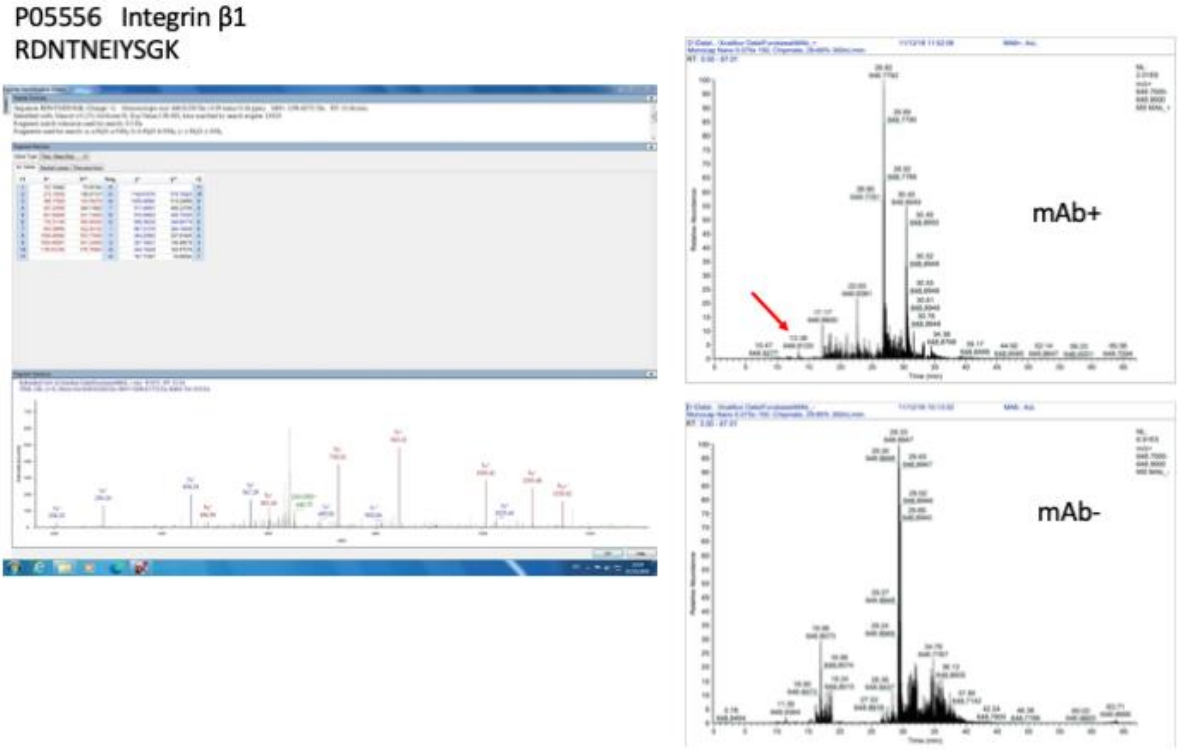

MS data of peptide, RDNTNEIYSGK
The peak of m/z 648.8129 corresponding to the peptide is found only in mAb+ sample (red arrow)

Supplementary Figure S3.'
MS data of peptide, RDNTNEIYSGK
The peak of m/z 648.8129 corresponding to the peptide is found only in mAb+ sample.

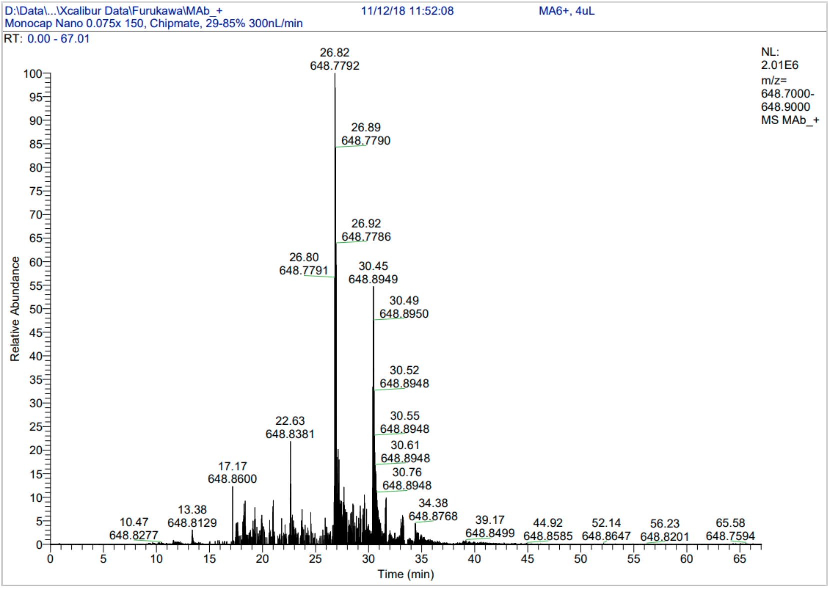

mAb+

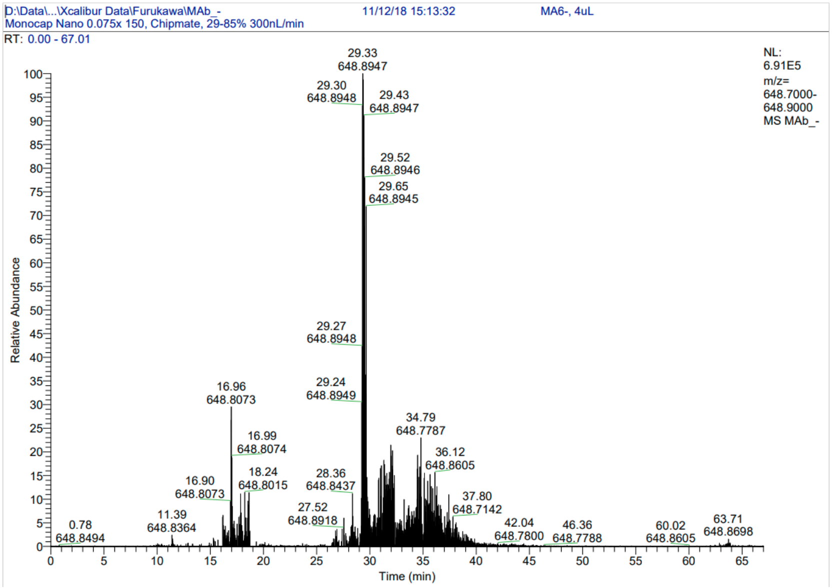

mAb-

Supplementary Figure S3.’’
P05556 Integrin β1
RDNTNEIYSGK   MS/MS data of the fragment ion

Peptide Identification Details

Peptide Summary

Sequence: RDNTNEIYSGK, Charge: +2, Monoisotopic m/z: 648.81250 Da (-0.09 mmu/-0.14 ppm), MH+: 1296.61772 Da, RT: 13.34 min,
Identified with: Mascot (v1.27); IonScore:31, Exp Value:2.5E-003, Ions matched by search engine: 13/120
Fragment match tolerance used for search: 0.5 Da
Fragments used for search: a; a-H_2O; a-NH_3; b; b-H_2O; b-NH_3; y; y-H_2O; y-NH_3

Fragment Matches

Value Type: Theo. Mass [Da]

Ion Series | Neutral Losses | Precursor Ions

#1	b^+	b^{2+}	Seq.	y^+	y^{2+}	#2
1	157.10840	79.05784	R			11
2	272.13535	136.57131	D	1140.51679	570.76203	10
3	386.17828	193.59278	N	1025.48984	513.24856	9
4	487.22596	244.11662	T	911.44691	456.22709	8
5	601.26889	301.13808	N	810.39923	405.70325	7
6	730.31149	365.65938	E	696.35630	348.68179	6
7	843.39556	422.20142	I	567.31370	284.16049	5
8	1006.45888	503.73308	Y	454.22963	227.61845	4
9	1093.49091	547.24909	S	291.16631	146.08679	3
10	1150.51238	575.75983	G	204.13428	102.57078	2
11			K	147.11281	74.06004	1

Fragment Spectrum

Extracted from: E:\Xcalibur Data\Furukawa\MA6_+.raw #1573 RT: 13.34
ITMS, CID, z=+2, Mono m/z=648.81250 Da, MH+=1296.61772 Da, Match Tol.=0.8 Da

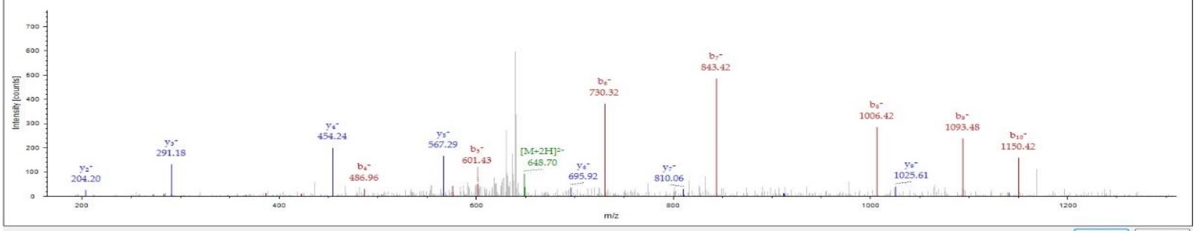

OK Help

Supplementary Figure S4.

Epidermal growth factor receptor(EGFR)

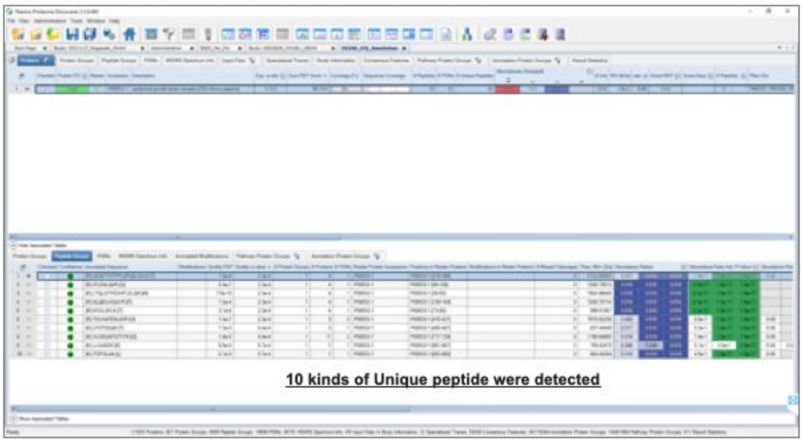

Ten peptide fragments of EGFR were detected.

Epidermal growth factor receptor(EGFR)

Cover Map

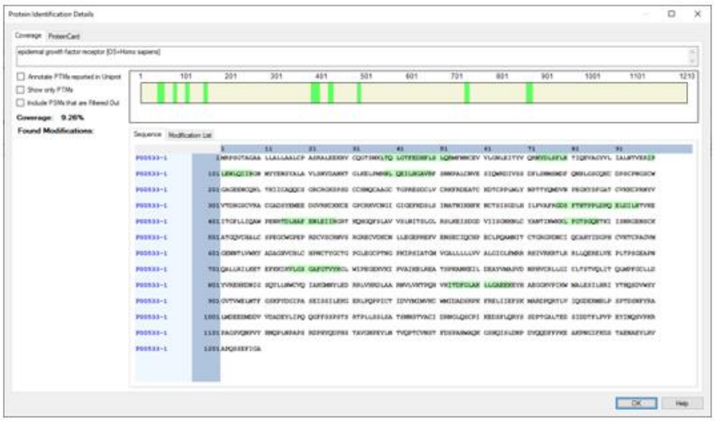

Distribution of 10 peptides of EGFR (cover map).

EGFR1

Epidermal growth factor receptor(EGFR)

Sequence: GDSFTHTPPLDPQELDILK, Charge: +3, Monoisotopic m/z: 708.35834 Da (-1.69 mmu/-2.39 ppm), MH+: 2123.06046 Da, RT: 51.9002 min,

#1	b+	b2+	b3+	Seq.	y+	y2+	y3+	#2
1	58.02874	29.51801	20.01443	G				19
2	173.05568	87.03148	58.35675	D	2066.04406	1033.52567	689.35287	18
3	260.08771	130.54749	87.36742	S	1951.01712	976.01220	651.01056	17
4	407.15613	204.08170	136.39023	F	1863.98509	932.49618	621.99988	16
5	508.20380	254.60554	170.07279	T	1716.91668	858.96198	572.97708	15
6	645.26272	323.13500	215.75909	H	1615.86900	808.43814	539.29452	14
7	746.31039	373.65884	249.44165	T	1478.81009	739.90868	493.60821	13
8	843.36316	422.18522	281.79257	P	1377.76241	689.38484	459.92565	12
9	940.41592	470.71160	314.14349	P	1280.70964	640.85846	427.57473	11
10	1053.49999	527.25363	351.83818	L	1183.65688	592.33208	395.22381	10
11	1168.52693	584.76710	390.18049	D	1070.57282	535.79005	357.52912	9
12	1265.57969	633.29348	422.53142	P	955.54587	478.27657	319.18681	8
13	1393.63827	697.32277	465.21761	Q	858.49311	429.75019	286.83589	7
14	1522.68086	761.84407	508.23181	E	730.43453	365.72090	244.14970	6
15	1635.76493	818.38610	545.92649	L	601.39194	301.19961	201.13550	5
16	1750.79187	875.89957	584.26881	D	488.30788	244.65758	163.44081	4
17	1863.87593	932.44161	621.96350	I	373.28093	187.14410	125.09850	3
18	1976.96000	988.98364	659.65818	L	260.19687	130.60207	87.40381	2
19				K	147.11280	74.06004	49.70912	1

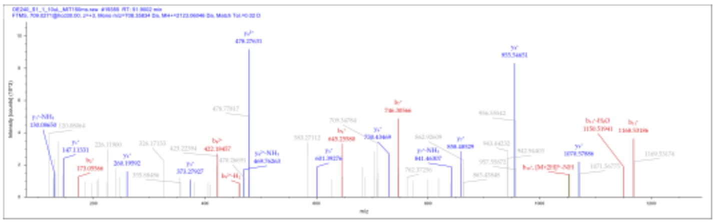

EGFR2

Epidermal growth factor receptor(EGFR)

Sequence: IPLENLQIIR, Charge: +2, Monoisotopic m/z: 604.87231 Da (+0.61 mmu/+1.01 ppm), MH+: 1208.73735 Da, RT: 48.1548 min

#1	b+	b2+	Seq.	y+	y2+	#2
1	114.09134	57.54931	I			10
2	211.14410	106.07569	P	1095.65207	548.32967	9
3	324.22817	162.61772	L	998.59931	499.80329	8
4	453.27076	227.13902	E	885.51524	443.26126	7
5	567.31369	284.16048	N	756.47265	378.73996	6
6	680.39775	340.70251	L	642.42972	321.71850	5
7	808.45633	404.73180	Q	529.34566	265.17647	4
8	921.54039	461.27384	I	401.28708	201.14718	3
9	1034.62446	517.81587	I	288.20302	144.60515	2
10			R	175.11895	88.06311	1

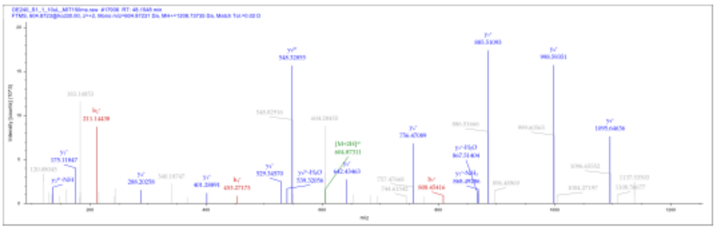

EGFR3

Epidermal growth factor receptor(EGFR)

Sequence: LTQLGTFEDHFLSLQR, Charge: +3, Monoisotopic m/z: 635.66742 Da (+0.41 mmu/+0.64 ppm), MH+: 1904.98771 Da, RT: 53.4879 min

#1	b+	b2+	b3+	Seq.	y+	y2+	y3+	#2
1	114.09134	57.54931	38.70196	L				16
2	215.13902	108.07315	72.38452	T	1791.90242	896.45485	597.97233	15
3	343.19760	172.10244	115.07072	Q	1690.85474	845.93101	564.28977	14
4	456.28166	228.64447	152.76540	L	1562.79617	781.90172	521.60357	13
5	513.30312	257.15520	171.77256	G	1449.71210	725.35969	483.90889	12
6	614.35080	307.67904	205.45512	T	1392.69064	696.84896	464.90173	11
7	761.41922	381.21325	254.47792	F	1291.64296	646.32512	431.21917	10
8	890.46181	445.73454	297.49212	E	1144.57455	572.79091	382.19637	9
9	1005.48875	503.24801	335.83444	D	1015.53196	508.26962	339.18217	8
10	1142.54766	571.77747	381.52074	H	900.50501	450.75614	300.83985	7
11	1289.61608	645.31168	430.54354	F	763.44610	382.22669	255.15355	6
12	1402.70014	701.85371	468.23823	L	616.37769	308.69248	206.13075	5
13	1489.73217	745.36972	497.24891	S	503.29362	252.15045	168.43606	4
14	1602.81623	801.91176	534.94360	L	416.26159	208.63444	139.42538	3
15	1730.87481	865.94104	577.62979	Q	303.17753	152.09240	101.73069	2
16				R	175.11895	88.06311	59.04450	1

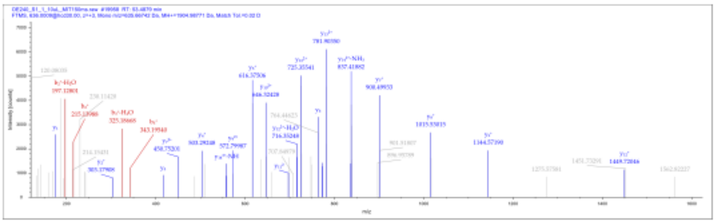

EGFR4

Epidermal growth factor receptor(EGFR)

ThermoFisher SCIENTIFIC

Sequence: NLQEILHGAVR, Charge: +3, Monoisotopic m/z: 417.23865 Da (+0.08 mmu/+0.2 ppm), MH+: 1249.70139 Da, RT: 40.8464 min

#1	b^+	b^{2+}	b^{3+}	Seq.	y^+	y^{2+}	y^{3+}	#2
1	115.05020	58.02874	39.02159	N				11
2	228.13427	114.57077	76.71627	L	1135.65822	568.33275	379.22426	10
3	356.19285	178.60006	119.40247	Q	1022.57415	511.79072	341.52957	9
4	485.23544	243.12136	162.41666	E	894.51558	447.76143	298.84338	8
5	598.31950	299.66339	200.11135	I	765.47298	383.24013	255.82918	7
6	711.40357	356.20542	237.80604	L	652.38892	326.69810	218.13449	6
7	848.46248	424.73488	283.49234	H	539.30486	270.15607	180.43980	5
8	905.48394	453.24561	302.49950	G	402.24594	201.62661	134.75350	4
9	976.52106	488.76417	326.17854	A	345.22448	173.11588	115.74634	3
10	1075.58947	538.29837	359.20134	V	274.18737	137.59732	92.06731	2
11				R	175.11895	88.06311	59.04450	1

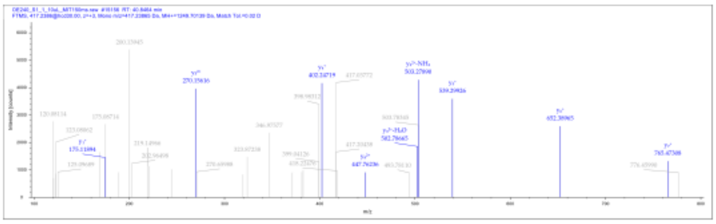

EGFR5

Epidermal growth factor receptor(EGFR)

ThermoFisher SCIENTIFIC

Sequence: NYDLSFLK, Charge: +2, Monoisotopic m/z: 500.26224 Da (+1.31 mmu/+2.63 ppm), MH+: 999.51720 Da, RT: 46.4604 min,

#1	b^+	b^{2+}	b^{3+}	Seq.	y^+	y^{2+}	y^{3+}	#2
1	115.05020	58.02874	39.02159	N				8
2	278.11353	139.56040	93.37603	Y	885.47165	443.23946	295.82873	7
3	393.14048	197.07388	131.71834	D	722.40832	361.70780	241.47429	6
4	506.22454	253.61591	169.41303	L	607.38137	304.19433	203.13198	5
5	593.25657	297.13192	198.42371	S	494.29731	247.65229	165.43729	4
6	740.32498	370.66613	247.44651	F	407.26528	204.13628	136.42661	3
7	853.40905	427.20816	285.14120	L	260.19687	130.60207	87.40381	2
8				K	147.11280	74.06004	49.70912	1

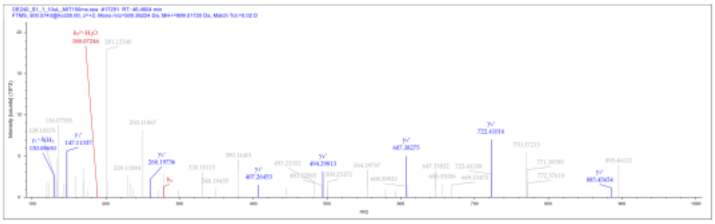

EGFR6

Epidermal growth factor receptor(EGFR)

ThermoFisher SCIENTIFIC

Sequence: TDLHAFENLEIIR, Charge: +3, Monoisotopic m/z: 524.27838 Da (-0.6 mmu/-1.14 ppm), MH+: 1570.82059 Da, RT: 49.7386 min

#1	b^+	b^{2+}	b^{3+}	Seq.	y^+	y^{2+}	y^{3+}	#2
1	102.05496	51.53112	34.68984	T				13
2	217.08190	109.04459	73.03215	D	1469.77470	735.39099	490.59642	12
3	330.16596	165.58662	110.72684	L	1354.74776	677.87752	452.25410	11
4	467.22487	234.11608	156.41314	H	1241.66370	621.33549	414.55942	10
5	538.26199	269.63463	180.09218	A	1104.60479	552.80603	368.87311	9
6	685.33040	343.16884	229.11498	F	1033.56767	517.28747	345.19407	8
7	814.37299	407.69014	272.12918	E	886.49926	443.75327	296.17127	7
8	928.41592	464.71160	310.14349	N	757.45666	379.23197	253.15707	6
9	1041.49999	521.25363	347.83818	L	643.41374	322.21051	215.14276	5
10	1170.54258	585.77493	390.85238	E	530.32967	265.66847	177.44808	4
11	1283.62664	642.31696	428.54707	I	401.28708	201.14718	134.43388	3
12	1396.71071	698.85899	466.24175	I	288.20302	144.60515	96.73919	2
13				R	175.11895	88.06311	59.04450	1

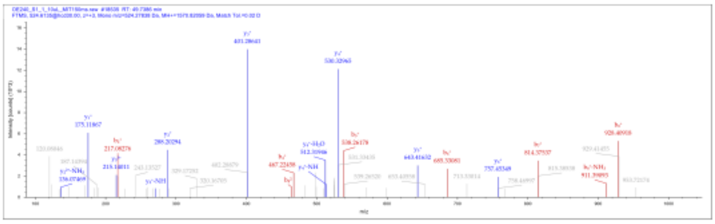

EGFR7

Epidermal growth factor receptor(EGFR)

Sequence: LFGTSGQK, Charge: +2, Monoisotopic m/z: 419.22693 Da (+0.04 mmu/+0.1 ppm), MH+: 837.44658 Da, RT: 21.2712 min

#1	b^+	b^{2+}	Seq.	y^+	y^{2+}	#2
1	114.09134	57.54931	L			8
2	261.15975	131.08352	F	724.36243	362.68485	7
3	318.18122	159.59425	G	577.29402	289.15065	6
4	419.22890	210.11809	T	520.27255	260.63991	5
5	506.26092	253.63410	S	419.22487	210.11608	4
6	563.28239	282.14483	G	332.19285	166.60006	3
7	691.34097	346.17412	Q	275.17138	138.08933	2
8			K	147.11280	74.06004	1

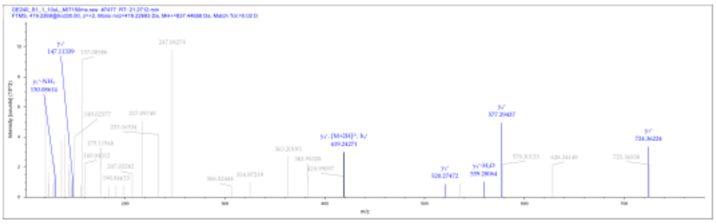

EGFR8

Epidermal growth factor receptor(EGFR)

Sequence: VLGSGAFGTVYK, Charge: +2, Monoisotopic m/z: 599.82794 Da (+0.98 mmu/+1.63 ppm), MH+: 1198.64861 Da, RT: 38.9445 min

#1	b^+	b^{2+}	Seq.	y^+	y^{2+}	#2
1	100.07569	50.54148	V			12
2	213.15975	107.08352	L	1099.57824	550.29276	11
3	270.18122	135.59425	G	986.49417	493.75072	10
4	357.21325	179.11026	S	929.47271	465.23999	9
5	414.23471	207.62099	G	842.44068	421.72398	8
6	485.27182	243.13955	A	785.41922	393.21325	7
7	632.34024	316.67376	F	714.38210	357.69469	6
8	689.36170	345.18449	G	567.31369	284.16048	5
9	790.40938	395.70833	T	510.29223	255.64975	4
10	889.47779	445.24254	V	409.24455	205.12591	3
11	1052.54112	526.77420	Y	310.17613	155.59170	2
12			K	147.11280	74.06004	1

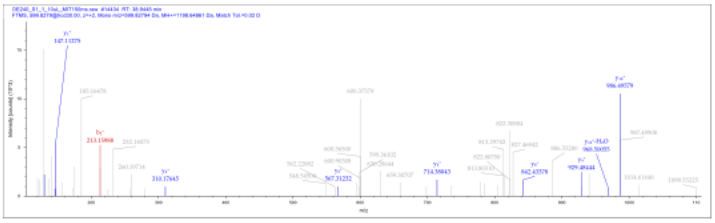

EGFR9

Epidermal growth factor receptor(EGFR)

Sequence: LLGAEEK, Charge: +2, Monoisotopic m/z: 380.21637 Da (+0.38 mmu/+1.01 ppm), MH+: 759.42546 Da, RT: 19.0507 min

#1	b^+	b^{2+}	Seq.	y^+	y^{2+}	#2
1	114.09134	57.54931	L			7
2	227.17540	114.09134	L	646.34063	323.67395	6
3	284.19687	142.60207	G	533.25657	267.13192	5
4	355.23398	178.12063	A	476.23510	238.62119	4
5	484.27657	242.64193	E	405.19799	203.10263	3
6	613.31917	307.16322	E	276.15540	138.58134	2
7			K	147.11280	74.06004	1

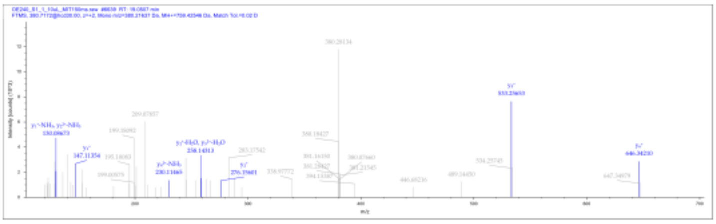

EGFR10

Epidermal growth factor receptor(EGFR)

Thermo Fisher SCIENTIFIC

Sequence: ITDFGLAK, Charge: +2, Monoisotopic m/z: 432.74478 Da (-0.13 mmu/-0.3 ppm), MH+: 864.48229 Da, RT: 33.7960 min

#1	b^{+}	b^{2+}	Seq.	y^{+}	y^{2+}	#2
1	114.09134	57.54931	I			8
2	215.13902	108.07315	T	751.39848	376.20288	7
3	330.16596	165.58662	D	650.35080	325.67904	6
4	477.23438	239.12083	F	535.32386	268.16557	5
5	534.25584	267.63156	G	388.25545	194.63136	4
6	647.33990	324.17359	L	331.23398	166.12063	3
7	718.37702	359.69215	A	218.14992	109.57860	2
8			K	147.11280	74.06004	1

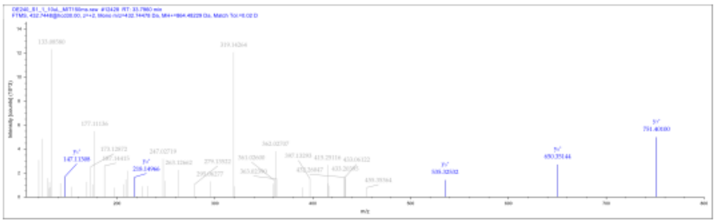

Focal adhesion kinase 1(FAK)

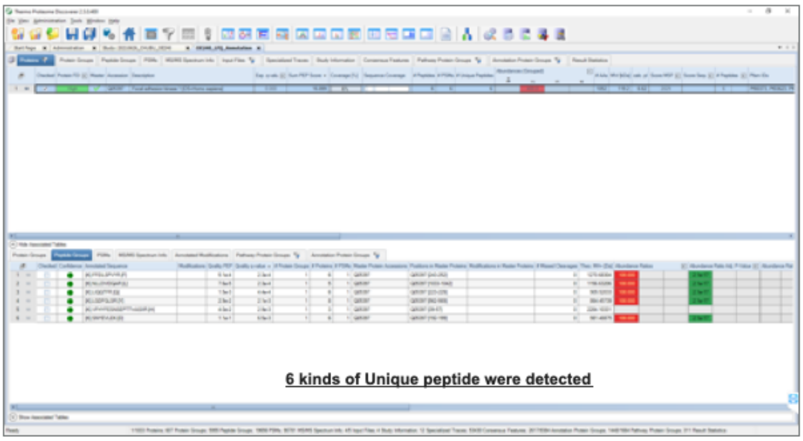

Six peptides of FAK were detected.

Focal adhesion kinase 1(FAK)

Cover Map

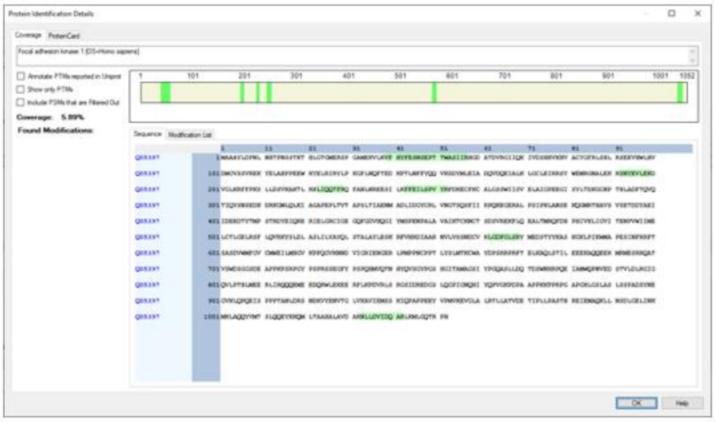

Distribution of 6 peptides of FAK (cover map) was shown.

FAK1

Focal adhesion kinase 1(FAK)

Sequence: FFEILSPVYR, Charge: +2, Monoisotopic m/z: 635.84534 Da (+0.18 mmu/+0.28 ppm), MH+: 1270.68340 Da, RT: 54.4557 min

#1	b^+	b^{2+}	Seq.	y^+	y^{2+}	#2
1	148.07569	74.54148	F			10
2	295.14410	148.07569	F	1123.61462	562.31095	9
3	424.18670	212.59699	E	976.54621	488.77674	8
4	537.27076	269.13902	I	847.50361	424.25545	7
5	650.35483	325.68105	L	734.41955	367.71341	6
6	737.38685	369.19707	S	621.33549	311.17138	5
7	834.43962	417.72345	P	534.30346	267.65537	4
8	933.50803	467.25765	V	437.25069	219.12899	3
9	1096.57136	548.78932	Y	338.18228	169.59478	2
10			R	175.11895	88.06311	1

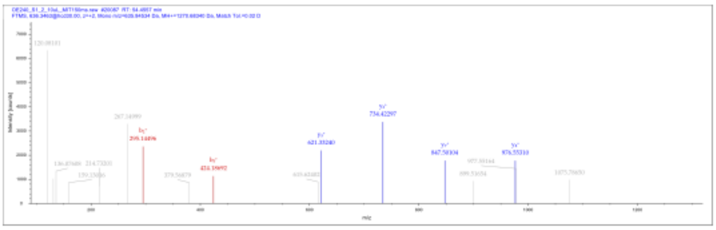

FAK2

Focal adhesion kinase 1(FAK)

Sequence: NLLDVIDQAR, Charge: +2, Monoisotopic m/z: 578.82007 Da (+0.4 mmu/+0.69 ppm), MH+: 1156.63286 Da, RT: 50.7300 min

#1	b^+	b^{2+}	Seq.	y^+	y^{2+}	#2
1	115.05020	58.02874	N			10
2	228.13427	114.57077	L	1042.58914	521.79821	9
3	341.21833	171.11280	L	929.50507	465.25617	8
4	456.24527	228.62628	D	816.42101	408.71414	7
5	555.31369	278.16048	V	701.39406	351.20067	6
6	668.39775	334.70251	I	602.32565	301.66646	5
7	783.42470	392.21599	D	489.24159	245.12443	4
8	911.48327	456.24527	Q	374.21464	187.61096	3
9	982.52039	491.76383	A	246.15607	123.58167	2
10			R	175.11895	88.06311	1

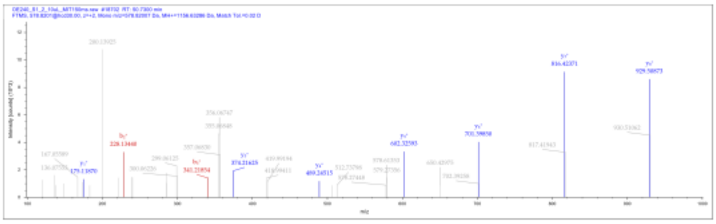

FAK3

Focal adhesion kinase 1(FAK)

Sequence: LIQQTFR, Charge: +2, Monoisotopic m/z: 453.26434 Da (+0.54 mmu/+1.2 ppm), MH+: 905.52141 Da, RT: 26.4026 min

#1	b^+	b^{2+}	Seq.	y^+	y^{2+}	#2
1	114.09134	57.54931	L			7
2	227.17540	114.09134	I	792.43626	396.72177	6
3	355.23398	178.12063	Q	679.35220	340.17974	5
4	483.29256	242.14992	Q	551.29362	276.15045	4
5	584.34024	292.67376	T	423.23504	212.12116	3
6	731.40865	366.20796	F	322.18737	161.59732	2
7			R	175.11895	88.06311	1

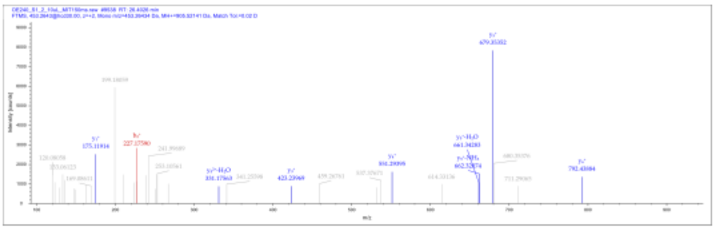

FAK4

Focal adhesion kinase 1(FAK)

ThermoFisher SCIENTIFIC

Sequence: LGDFGLSR, Charge: +2, Monoisotopic m/z: 432.73233 Da (0 mmu/-0.01 ppm), MH+: 864.45738 Da, RT: 35.0126 min

#1	b^+	b^{2+}	Seq.	y^+	y^{2+}	#2
1	114.09134	57.54931	L			8
2	171.11280	86.06004	G	751.37333	376.19030	7
3	286.13975	143.57351	D	694.35187	347.67957	6
4	433.20816	217.10772	F	579.32492	290.16610	5
5	490.22962	245.61845	G	432.25651	216.63189	4
6	603.31369	302.16048	L	375.23504	188.12116	3
7	690.34572	345.67650	S	262.15098	131.57913	2
8			R	175.11895	88.06311	1

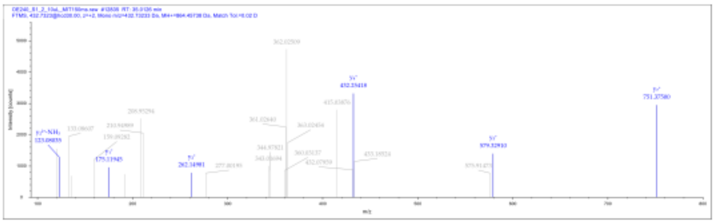

FAK5

Focal adhesion kinase 1(FAK)

ThermoFisher SCIENTIFIC

Sequence: VFHYFESNSEPTTWASIIR, Charge: +3, Monoisotopic m/z: 762.04608 Da (+6.8 mmu/+8.92 ppm), MH+: 2284.12369 Da, RT: 53.5452 min

#1	b^+	b^{2+}	b^{3+}	Seq.	y^+	y^{2+}	y^{3+}	#2
1	100.07569	50.54148	34.03008	V				19
2	247.14410	124.07569	83.05289	F	2185.03489	1093.02109	729.01648	18
3	384.20302	192.60515	128.73919	H	2037.96648	1019.48688	679.99368	17
4	547.26634	274.13681	183.09363	Y	1900.90757	950.95742	634.30737	16
5	694.33476	347.67102	232.11644	F	1737.84424	869.42576	579.95293	15
6	823.37735	412.19231	275.13063	E	1590.77583	795.89155	530.93013	14
7	910.40938	455.70833	304.14131	S	1461.73323	731.37025	487.91593	13
8	1024.45231	512.72979	342.15562	N	1374.70120	687.85424	458.90525	12
9	1111.48434	556.24581	371.16630	S	1260.65828	630.83278	420.89094	11
10	1240.52693	620.76710	414.18049	E	1173.62625	587.31676	391.88027	10
11	1337.57969	669.29348	446.53142	P	1044.58366	522.79547	348.86607	9
12	1438.62737	719.81732	480.21397	T	947.53089	474.26908	316.51515	8
13	1539.67505	770.34116	513.89653	T	846.48321	423.74525	282.83259	7
14	1725.75436	863.38082	575.92297	W	745.43554	373.22141	249.15003	6
15	1796.79148	898.89938	599.60201	A	559.35622	280.18175	187.12359	5
16	1883.82350	942.41539	628.61269	S	488.31911	244.66319	163.44455	4
17	1996.90757	998.95742	666.30737	I	401.28708	201.14718	134.43388	3
18	2109.99163	1055.49945	704.00206	I	288.20302	144.60515	96.73919	2
19				R	175.11895	88.06311	59.04450	1

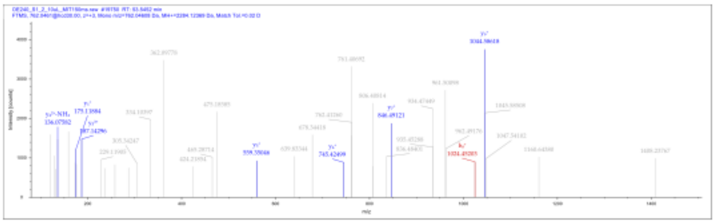

9

FAK6

Focal adhesion kinase 1(FAK)

ThermoFisher SCIENTIFIC

Sequence: SNYEVLEK, Charge: +2, Monoisotopic m/z: 491.24835 Da (+0.34 mmu/+0.69 ppm), MH+: 981.48943 Da, RT: 26.2557 min

#1	b^+	b^{2+}	Seq.	y^+	y^{2+}	#2
1	88.03930	44.52329	S			8
2	202.08223	101.54475	N	894.45672	447.73200	7
3	365.14556	183.07642	Y	780.41380	390.71054	6
4	494.18815	247.59772	E	617.35047	309.17887	5
5	593.25657	297.13192	V	488.30788	244.65758	4
6	706.34063	353.67395	L	389.23946	195.12337	3
7	835.38322	418.19525	E	276.15540	138.58134	2
8			K	147.11280	74.06004	1

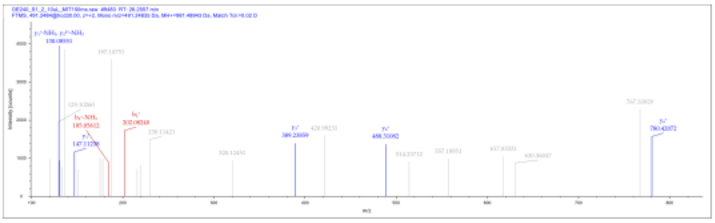

Supplement: Supplementary file 1 [file ijms-23-00423-s001.zip › ijms-1464716-supplementary.pdf]
